# Feel what you read: Specific aspects of empathy modulate semantic retrieval processes and representational content of emotion-label, emotion-laden, and neutral abstract words

**DOI:** 10.1371/journal.pone.0341113

**Published:** 2026-01-20

**Authors:** Miriam Rademacher, Linda Espey, Marta Ghio, Laura Bechtold

**Affiliations:** Department of Biological Psychology, Institute of Experimental Psychology, Faculty of Mathematics and Natural Sciences, Heinrich Heine University, Düsseldorf, Germany; University of Maribor, SLOVENIA

## Abstract

Building on evidence for experience-specific grounding of word meaning and interindividual differences therein, this study investigated how specific aspects of empathy modulate the processing and representation of abstract emotional words. We investigated single-trial N400 amplitudes as a measure of semantic retrieval in 78 healthy adults during a delayed lexical decision task with emotion-label, emotion-laden, and neutral abstract words. We further measured the participants’ levels of empathic concern, fantasy, personal distress, and perspective taking. Additionally, ratings on valence, arousal, and emotional experience quantified the words’ emotional representational content. While direct comparison yielded no evidence for N400 differences between word types, N400 amplitudes in response to emotion-label words decreased with increasing fantasy scores, with this modulation being stronger than for emotion-laden and neutral words. Additionally, participants with higher fantasy scores rated emotional words higher in absolute valence. The observed N400 reductions thus seem to reflect fantasy-driven processing facilitation graded by the words’ emotionality level. In contrast, we found no evidence for N400 modulations by empathic concern, personal distress, or perspective taking while affective ratings on all scales increased with increasing empathic concern scores. Our findings suggest that fantasy facilitates emotion-label word processing, and empathic concern enriches emotional word meaning representations, demonstrating interindividual differences in the experiential grounding of emotional abstract concepts.

## Introduction

Conceptual representations of word meanings integrate all information derived from the experiences we gain about a word’s referent in our semantic memory [1]. The theory of *grounded cognition* [2] assumes that cognition in general (and the processing of word meaning specifically) relies on widespread reactivations of the respective experience-specific brain areas. Thus, during the processing of word meaning, experiential brain regions are reactivated and higher-order brain areas combine the provided information forming the neural representation of a word’s meaning [3]. The quality as well as quantity of underlying experiential information modulate this integrative semantic retrieval process, affecting neural activation and behavioral performance [4].

The grounding of distinct word processing stages can be investigated via *electroencephalography* [5]. Regarding semantic retrieval and integration of conceptual information, the *N400*, peaking negatively at around 300–500 ms, is the central component of interest in the *event-related potential* [ERP; for a review, see 6]. One long standing finding is that the N400 is sensitive to the concreteness of words, i.e., to what extent words refer to something that is perceivable via the external modalities (sight, hearing, etc.). Concrete words have been shown to elicit larger N400 amplitudes than abstract words [7–11]. This has been interpreted to reflect the relatively richer, multimodal conceptual representations of concrete compared to abstract words [12]. Lateralized concreteness effects over the right hemisphere have been interpreted to reflect the external perceptual qualities of these enriching experiences in contrast to rather left-lateralized language-based enrichment [9]. While contrasting concrete and abstract words delivered important insights into experience-driven processing differences, more fine-grained categories within the concrete domain have deepened our understanding of grounded word processing. Now, probing the generalizability of grounding mechanisms to the abstract domain is a crucial and still ongoing step towards a comprehensive understanding of grounded semantic cognition [for a discussion, compare 13,14].

Within the abstract domain, emotional words, such as *joy* or *failure*, form a clearly defined subcategory suitable to investigate experience-specific grounding [14,15]. Psycholinguistic rating studies identified valence and arousal to be crucial dimensions to differentiate emotional from neutral abstract words [16, for a discussion, see 17]. At the behavioral level, many studies report an *emotionality effect* – that is, a robust processing advantage for emotional words, reflected in faster reaction times relative to neutral words [for a review, see 14]. At the electrophysiological level, emotional words elicit reduced N400 amplitudes compared to neutral words [18,19]. In line with the behavioral processing advantage, this N400 reduction has been interpreted to reflect a prioritized and facilitated processing of affective stimuli [18–20]. Notably, effects of concreteness and emotionality – although both reflect a relative enrichment and result in faster response times – elicit opposite N400 modulations, which points towards a dissociation of the behavioral versus electrophysiological level effects [10].

Emotional words can and should be further subdivided into *emotion-label* and *emotion-laden* words [14,21]. Emotion-label words, like *joy* or *sorrow*, directly refer to a distinct emotion, while emotion-laden words, like *friendship* or *failure,* indirectly refer to emotional experience through emotional connotation. Please note that emotion-laden words can also be concrete (e.g., *bomb* or *puppy*), which makes it vital to control for concreteness in the comparison of emotion-label and emotion-laden words to avoid confounds. Betancourt, Guasch and Ferré [16] showed that valence and arousal do not differentiate between emotion-label and emotion-laden words. Rather, they are differentiated by multidimensional emotional experiences including interoception and the subjective feeling, which integrates cognitive and physical experiences. Some studies have demonstrated a behavioral processing advantage for emotion-label over emotion-laden words by measuring response times in implicit [e.g., lexical decision tasks; 19,22,23,24] and explicit processing tasks [e.g., categorization tasks; 25,26], while other studies did not find evidence for such a fine-grained emotionality effect in an implicit lexical decision task [26–29].

Electrophysiological investigations might be able to shed light onto whether and at which stage emotion-label and emotion-laden word processing differ. However, evidence is still sparse and paradigms differ largely: Two ERP studies employing different tasks reported a reduced N400 in response to emotion-label compared to emotion-laden words, which was interpreted to reflect a relatively stronger processing facilitation in line with the emotionality effect [30] (flanker task), [31] (emotional categorization task)]. Another study did not find evidence for an N400 amplitude difference between emotion-label and emotion-laden words in a lexical decision task [19], with a potentially underpowered analysis due to the small sample size of only 23 participants. In contrast, a study with an emotional Stroop task reported an enhanced N400 in response to emotion-label compared to emotion-laden words [32]; however, this finding was probably driven by a conflict between color information and affective information, which was larger for emotion-label words due to their direct and thus salient reference to emotions. Further, few studies have reported lateralized effects, and those involved ERP components other than the N400: One study found a higher P1 (reflecting early attentional allocation to emotional words) for emotion-laden compared to emotion-label words over the right versus left hemisphere [32], while another found a right hemispheric preferential processing of emotion-label words in the N170 (reflecting early sensitivity to emotional content) and the late positive component [i.e., a positive deflection between 500 and 700 ms, reflecting sustained emotional evaluation; 24]. Hence, whether and how the processing stage of semantic retrieval reflected by the N400 is grounded in the experience related to a word’s emotionality (i.e., emotion-label versus emotion-laden versus neutral) has still not been clarified. Of potential interest in order to be able to contextualize previous contradictory effects are recent theoretical developments, which stress the importance of *interindividual differences* in grounded cognition, as such differences can lead to heterogeneous findings when not accounted for [33].

With respect to emotional experience, trait *empathy*, i.e., the ability to correctly interpret the emotional state of others, seems a promising candidate to shape emotional grounding. It has been shown that higher empathy increases the perceived arousal and intensity of negative and positive emotional sentences, respectively, leaving neutral sentences unaffected [34]. Additionally, higher empathy seems to enhance emotional word processing by improving the perception and appraisal of emotional content [35], facilitating efficient emotional word comprehension [36], and enabling rapid top-down language processing using social cues [37]. Hinting at the neural origins of this effect, empathic processes seem to share neural substrates with the emotion processing network [38,39]. Further, there is evidence that empathic processes activate specifically the right hemisphere [40]. A behavioral study recently reported first evidence for a graded empathy-driven modulation of lexical decision response times that was stronger for emotion-label than for emotion-laden than for neutral abstract words [29]. However, to the best of our knowledge, no study so far has aimed to provide empirical electrophysiological evidence for empathy-related processing differences between emotion-label, emotion-laden, and neutral abstract words.

Empathy appears to be a multifaceted construct and different aspects of empathy might affect different cognitive processes [41]. A well-validated and frequently used tool to measure empathy, the *Interpersonal Reactivity Index* [IRI; 42], does indeed appear to measure interrelated but clearly delineated aspects of empathy with its four subscales: i) *empathic concern* measures prosocial sympathy and concern, ii) *fantasy* measures imaginative immersion in the feelings of fictional characters, iii) *personal distress* measures self-oriented negative feelings in unpleasant social situations, and iv) *perspective taking* measures the tendency to adopt another person’s psychological perspective. In an approach to reduce complexity, these scales have been assigned to the higher-level aspects of cognitive versus emotional empathy, but there are clear recommendations to study the four scales separately [41,43]. Crucially, certain empathy aspects might play a larger role in driving interindividual differences in emotional word processing than others: The emotional responsiveness involved in empathic concern and the tendency to mentally simulate emotional experience involved in fantasy seem most likely to affect the experience involved in – and thus the grounding of – emotional concepts. Processes involved in personal distress (i.e., poor emotional regulation) and perspective taking (i.e., rather purely cognitive mentalizing) should play less of a role in this context.

The current study investigated whether semantic retrieval processes and emotionality-derived representational content differ between emotion-label, emotion-laden, and neutral abstract words and whether these differences are further modulated by specific aspects of empathy. Therefore, we measured ERPs during a delayed lexical decision task, i.e., an implicit word (versus pseudoword) recognition task that is widely used [19,22–24]. Crucially, the implicitness of the lexical decision task excludes epiphenomenal confounds that could potentially be introduced by more explicit tasks [14,44]. Additionally, we collected information on the participants’ empathy with the German translation of the IRI, the *Saarbrücker Persönlichkeitsfragebogen* [SPF; 43] on the subscales empathic concern, fantasy, personal distress, and perspective taking. Using separate linear mixed effects (LME) analyses per subscale, we investigated how these aspects of empathy modulate left- and right-hemispheric single-trial N400 amplitudes elicited by emotion-label, emotion-laden, and neutral abstract words. We further explored how the empathy measures modulate the words’ emotional representational content. We therefore collected word ratings on valence, arousal, and association with emotional experience from the same participants who did the lexical decision task.

We expected word emotionality to gradually reduce the N400 with emotion-label words showing the least negative N400, followed by emotion-laden and neutral words. More crucially, we expected an interaction between word emotionality and specific aspects of empathy. Specifically, we expected more distinct effects and a higher explanatory power for both the empathic concern and fantasy subscales compared to the perspective taking and personal distress subscales. We expected higher levels of empathic concern and fantasy to reduce N400 amplitudes more strongly for emotion-label than for emotion-laden than for neutral words. These effects could be more pronounced over the right hemisphere, which we assume to be more strongly involved in emotional and empathetic processes. Regarding the ratings, we expected more extreme (positive or negative) valence and higher arousal ratings for emotional (emotion-label and emotion-laden) than for neutral words and a fully graded pattern (emotion-label > emotion-laden > neutral) for emotional experience ratings. Higher levels of empathic concern and fantasy should further magnify these effects by increasing the rating values specifically for emotional words.

## Materials and methods

### Sample

In total, 91 volunteers were recruited to take part in this study via flyers at the university and social media between 20/03/2023 and 04/04/2024. We excluded four participants due to a reported psychiatric medical history, one participant due to ambidexterity, and one participant due to low compliance (random response pattern). Seven participants had to be excluded due to excessive technical or muscle artifacts in the EEG signal (leading to a loss of more than 25% of trials per participant). All remaining 78 participants (62 female, 16 male, 0 diverse) were healthy, right-handed German native speakers, had normal or corrected-to-normal vision, and had at least a university entrance degree. Their age ranged from 18 to 31 years (*M* = 22.46 years, *SD* = 3.15 years). Two further participants had to be excluded only from the analyses including valence ratings as dependent variable and covariate due to missing data or misunderstood instructions for the bipolar valence scale. Participants received either course credit or monetary compensation. All participants were informed about voluntariness and gave written informed consent prior to participating. The study fulfilled the requirements of the declaration of Helsinki and was approved by the ethics committee of the Faculty of Mathematics and Natural Sciences of Heinrich Heine University Düsseldorf.

### Material

The stimuli consisted of 180 abstract nouns, including 60 emotion-label, 60 emotion-laden, and 60 neutral abstract words. Only emotion-label words were classified as *feeling* in the GermaNet database [45]. The words were selected from a pool of 329 abstract nouns based on pre-experimental ratings provided by German native speakers. To minimize response bias, 20 concrete filler words were included in the pre-experimental ratings. Participants in the pre-experimental ratings rated the words on Likert-type scales assessing concreteness (*not at all* [1] to *very concrete* [5]), valence (*very negative* [−4] to *very positive* [4]), and arousal (*not at all* [1] to *very strongly* [9] *associated with arousal*). All 329 words in the initial pool were abstract, i.e., received concreteness ratings between 1 and 3. Based on the valence ratings, we further subdivided the stimuli into negative (< −1), neutral (from −1 to 1), and positive (> 1).

Emotion-label and emotion-laden words consisted of 30 positive and 30 negative words, each. Emotion-label, emotion-laden, and neutral words did not differ in their signed valence (confirmed by independent *t*-tests, *p* ≥ .512 [uncorrected] for all pairwise comparisons), which was neutral on average. Positive emotion-label, negative emotion-label, positive emotion-laden, and negative emotion-laden words were additionally matched for absolute valence and arousal (confirmed by independent samples *t*-tests, *p* ≥ .215 [uncorrected] for all pairwise comparisons). Positive emotion-label, negative emotion-label, positive emotion-laden, negative emotion-laden, and neutral words were between 4–14 letters long and were further matched regarding spoken [SUBTLEX; 46] and written [CELEX; 47] word frequency, and concreteness (confirmed by independent samples *t*-tests, *p* ≥ .233 [uncorrected] for all pairwise comparisons). For descriptive statistics, see Table 1.

**Table 1:** Psycholinguistic properties of the words based on the pre-experimental ratings.

Variable	Emotion-label				Emotion-laden				Neutral^b^	
	Negative^a^		Positive^a^		Negative^a^		Positive^a^			
	*M*	*SD*	*M*	*SD*	*M*	*SD*	*M*	*SD*	*M*	*SD*
length	8.27	2.75	9.53	2.75	8.13	2.58	9.40	2.28	8.42	2.33
written frequency	6.60	7.31	12.20	23.50	8.97	15.14	10.50	19.96	11.83	19.13
spoken frequency	14.91	34.50	14.53	25.22	17.96	42.64	10.74	18.42	11.38	20.56
concreteness	1.91	0.23	1.92	0.28	1.92	0.33	1.95	0.48	1.98	0.43
arousal	6.15	1.01	6.13	1.18	6.13	1.02	5.83	0.85	3.37	0.94
signed valence	−2.35	0.47	2.37	0.68	−2.46	0.60	2.33	0.57	0.18	0.37

*M* = mean, *SD* = standard deviation.

^a^*n* = 30

^b^*n* = 60

Additionally, 180 pseudowords were created for the lexical decision task with the pseudoword generator *Wuggy* [German language module; 48]. They were matched with the real words for overall length, length of subsyllabic segments, and transition frequencies between letters. The thereby ensured similarity to real German words aimed to maximize potential processing differences [49]. Visual inspection assured that pseudowords did not contain words or word fragments that resembled German words. A full list of stimuli, including the pre-experimental ratings of the words, is available in the OSF repository: https://osf.io/pe84t/.

### Procedure

Data acquisition took place in an EEG-laboratory at the university with one participant at a time. Before entering the experimental procedure, each participant was informed about voluntariness and data protection. They gave written informed consent before filling out the digital demographic questionnaire. Afterwards, the EEG was set up. Following a standardized protocol, each participant received instructions about how to perform the lexical decision task and avoid artifacts: they were asked to sit still, fixate the fixation cross, and make their lexical decisions on the (pseudo)words as quickly and accurately as possible, as soon as the response-key assignment was displayed. Participants responded with their right or left index finger, which should be kept on the right and left Ctrl-key of a USB keyboard, respectively, throughout the experiment.

**Fig 1:**
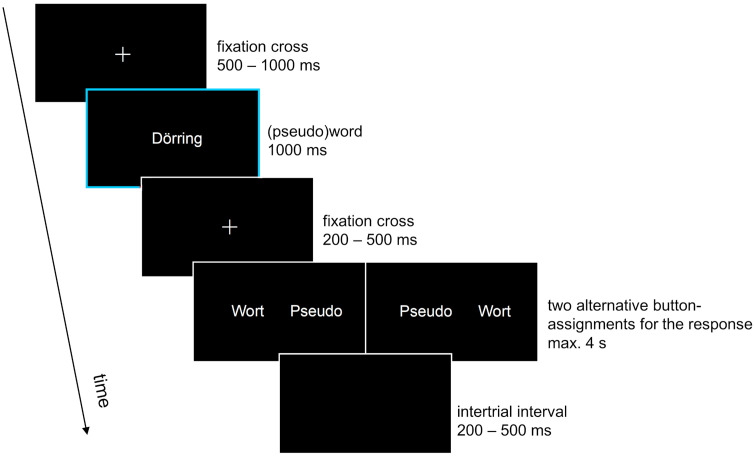
Sequence of events in the experimental trials. (Pseudo)word presentation (marked in blue) was the event of interest for the EEG analysis. Responses were delayed and the two possible response-button-assignments counterbalanced to keep the time window following word presentation (highlighted in blue) free from motor (preparation) artifacts.

Participants completed 12 practice trials including all experimental conditions (two emotion-label, two emotion-laden, two neutral words, six pseudowords, neither of which were used in the actual experiment). Then, they completed the 360 experimental trials (60 emotion-label, 60 emotion-laden, 60 neutral, 180 pseudowords, presented in randomized order). As displayed in Fig 1, each trial started with a centrally presented fixation cross for a random interval between 500–1000 ms, followed by the (pseudo)word for 1000 ms, another fixation cross for a random interval between 200–500 ms, a response screen for a maximum of 4 s, and lastly an intertrial interval showing a blank screen for a random interval between 200–500 ms. The response screen showed the words “pseudo” and “word” displayed randomly either on the left or on right side of the screen to prevent action preparation during the preceding presentation of the (pseudo)word. Random intervals and delayed responses aimed to minimize motor (preparation) artifacts. If participants did not respond within 4 s, a prompt instructing them to respond faster was displayed, after which the next trial started. Participants could take a break every 30 trials, the length of which was determined by the participants. The duration of the lexical decision task was about 20 minutes.

The lexical decision task was run by the software *Presentation* (Version 22.0, Neurobehavioral Systems, Inc., Berkeley, CA, www.neurobs.com) and all text was displayed in white letters on a black background in Arial font (30 pt). The whole experiment was conducted on a Windows 10 Silverstone PC with a 27” BenQ LCD HDMI Monitor (1920 × 1080-pixel resolution, 60 Hz refresh rate) and a USB keyboard.

After EEG recording, participants were instructed to fill out the SPF and the Vividness of Emotional Imagery Questionnaire, for which we alternated the order between participants. The latter questionnaire measures how vividly participants can imagine emotions on 5-point Likert-type scales ranging from (“I am not feeling the emotion, I only think about it”) to 5 (“I feel the emotion very strongly”) based on 12 emotional adjectives (e.g., angry or excited; [50]). Both questionnaires were programmed with *Python*, showing first the instructions with examples, and then eight items per page for the SPF (six for the Vividness of Emotional Imagery Questionnaire). Only the SPF was used to derive predictor variables entered into the analyses conducted in this study.

Questionnaires were followed by the word ratings. Ratings were collected on three Likert scales including association with *emotional experience* and *arousal* (from 1 *not at all*, to 9 *very strongly*) as well as on a bipolar scale for *valence* (from −4 *very negative*, over 0 *neutral* to +4 *very positive*). Participants received standardized instructions asking them to enter their spontaneous and subjective rating using the whole range of the scale and informing them that there were no correct or wrong answers. The word ratings were conducted on *SoSciSurvey* (https://www.soscisurvey.de) and included the same 180 abstract nouns used in the lexical decision task plus an additional 20 concrete words to avoid biased ratings (total of 200 words). For each rating scale, the 200 words were divided onto four pages of 50 words each, and on each page, each word was displayed on a separate row in black letters (font: Arial) against a white background. We randomized the order of the rating scales, as well as the page order within each scale and the order of words per page. Post-experimental questionnaires and ratings took about 50 minutes. All in all, the data acquisition took about two hours.

### EEG acquisition and preprocessing

The EEG was set up with the 28 Ag/AgCl active electrodes mounted on a textile *ActiCap* (Brain Products GmbH) according to the extended international 10–20 system [51] at sites F7, F3, Fz, F4, F8, FC5, FC1, FC2, FC6, T7, C3, Cz, C4, T8, CP5, CP1, CP2, CP6, P7, P3, Pz, P4, P8, PO9, O1, Oz, O2, and PO10. The ground electrode was positioned at AFz and the online reference at FCz. Four additional electrodes were attached, one to each mastoid as later offline reference, one to the outer corner of the left eye and one above the left eye (site FP2) to record the horizontal and vertical electrooculogram, respectively. Impedances were kept below 20 kΩ. EEG was recorded using the *BrainVision Recorder* software (version 1.20.0506) and the *BrainAmp DC amplifier* (Brain Products GmbH) with an online sampling rate of 1000 Hz and no additional online filters.

We analyzed the EEG data using the *Brain Vision Analyzer* (Version 2.2; Brain Products GmbH). For 12 participants, one electrode out of the further analyzed electrodes (i.e., either F3, F4, FC5, FC1, FC2 or FC6, as specified in *Statistical Analysis* below) had to be pooled with equidistantly surrounding (two to four) electrodes due to extensive technical artifacts. Data were re-referenced on the averaged mastoids or – in case of seven participants – only one mastoid (left mastoid only: three participants, right mastoid only: four participants) due to continuous muscle artifacts in the other reference channel. We then applied a Butterworth zero phase shift filter with a low cutoff of 0.1 Hz (time constant: 1.59), a high cutoff of 30 Hz, and a notch filter of 50 Hz. A fast, restricted independent component analysis with classical sphering identified independent components based on an interval starting at 60 s after recording start and a length of 120 s. The components were then visually inspected to detect blink artifacts (i.e., frontally pronounced sharp positive deflections with a frontal pronunciation, co-occurring with deflections in the electrooculogram). After exclusion of the components containing the blink artifacts, the signal was reconstructed via an inverse independent component analysis. The data was then segmented from 400 ms before to 1200 ms after the word onset and we conducted a baseline correction with the 200 ms interval before the word-onset.

To improve the signal to noise ratio, we conducted an automatic artifact rejection with a maximal allowed voltage step of 50 µV/ms, a maximal allowed difference of values in intervals of 100 µV per 100 ms, a minimal allowed amplitude of −75 µV and a maximum allowed amplitude of 75 µV, and a lowest allowed activity of 0.1 µV per 100 ms. For statistical analysis, single-trial segments were generically exported, then averaged over the time frame of interest, and combined with the behavioral data using *MATLAB* (version R2021a; full data set available in the OSF repository: https://osf.io/pe84t/). For visual display, the data was averaged separately for emotion-label, emotion-laden, neutral, and pseudowords per participant and then further averaged across participants per word type to generate grand averages.

### Statistical analysis

All statistical analyses were performed with *R* (version 4.3.1) in the *RStudio* environment (version 2023.09.1). All analysis scripts are available in the OSF repository: https://osf.io/pe84t/. We conducted mixed linear effects (LME) analyses in order to test our hypotheses regarding effects on single-trial N400 amplitudes as well as absolute valence, arousal, and emotional experience rating values. We opted for LME analyses as they allowed us to include categorical and continuous fixed-effects predictors and to control for unsystematic variance introduced by subjects, words, and electrodes through random effects [52,53]. We used the R packages *tidyverse* [version 2.0.0; 54], *dplyr* [version 1.1.3; 55], and *stringr* [version 1.5.0; 56] for data handling. The R packages *lme4* [version 1.1−34; 57], *lmerTest* [version 3.1−3; 58], and *interactions* [version 1.1.5; 59] were used for LME analysis, and *ggplot2* [version 3.4.4; 60] and *ggpubr* [version 0.6.0; 61] for visualization.

### N400 LME analyses

The single-trial N400 amplitude was defined as the mean amplitude from 300 ms to 450 ms after word onset and was measured at frontocentral electrodes sites F3, F4, FC5, FC1, FC2, FC6. The time window and topography were chosen in line with the relevant literature [e.g., 19,20] and based on the visual inspection of grand averages. The grand average shows a clear negative deflection and the well-known frontocentral word-pseudoword N400 effect in this time window [see, e.g., 62]. Word-trials with incorrect lexical decisions were excluded prior to analysis (1650 data points out of 78780 data points, ~ 2% of the data).

We set up one LME model for each SPF subscale. In each model, we included emotionality as a three-level fixed-effect factor (emotion-label, emotion-laden, neutral; within-subjects). Two contrast matrices were set up to allow all three pairwise comparisons of the factor levels: one with neutral as reference condition (i.e., emotion-label versus neutral and emotion-laden versus neutral), one with emotion-label as reference condition (adding the comparison emotion-laden versus emotion-label). Further, we included electrode laterality as a categorical fixed-effect predictor (left hemisphere including F3, FC1, and FC5 [−0.5] versus right hemisphere including F4, FC2, and FC6 [0.5]; within-subjects). The between-subject measure of the respective SPF subscale score was included as mean-centered and normalized continuous fixed-effect predictor, separately for each of the SPF subscales: i) empathic concern, ii) fantasy, iii) perspective taking, and iv) personal distress (see Table 2 for descriptive statistics on subscale scores).

**Table 2:** Descriptive data of SPF subscale scores.

Subscale	*M*	*SD*	Min	Max
Empathic Concern	16.10	2.36	10	20
Fantasy	14.97	3.27	7	20
Personal Distress	11.23	3.00	6	19
Perspective Taking	15.24	2.50	8	20

SPF = Saarbrücker Persönlichkeitsfragebogen; *M* = mean, *SD* = standard deviation, Min = minimum score; Max = maximum score (scores could vary from 1 to 20 for each scale).

Regarding our random effect structure, we included a random intercept and slope per subject, word, and electrode in each LME model. This resulted in the following model terms for the N400 LME analyses (with “empathy measure” standing for one of the four SPF subscale scores, respectively):


N400 ~ emotionality * laterality * empathy measure + (1|subject) + (1|word) + (1|electrode)

We refrained from including a more complex random effect structure, i.e., additional random slopes of emotionality and/or laterality (and their interaction) per subject as well as empathy and/or laterality (and their interaction) per word. As our hypotheses centrally assumed systematic (rather than random) between-subject and between-word variance to be explained in interactions with the respective within-subject (emotionality, laterality) and between-subject predictors (empathy), including the random slopes would have entailed the risk of violating the assumption of independence of fixed and random effects, i.e., resulted in endogeneity [63].

For outlier correction, we excluded data points with residuals that deviated by more than 2.5 standard deviations from the residual mean after first fit of each model [64], respectively (see Table 3 for the number of data points included in the LME analyses). We then refitted the respective LME model in order to investigate main and interaction effects of our fixed-effect predictors. We further looked at planned contrasts for significant effects involving the emotionality factor to identify the factor-levels differing significantly from each other and conducted simple slope analyses to resolve significant interaction effects. To fully explore interactions of empathy measures with emotionality in the N400 LME analyses, we additionally examined the significance of the emotionality effect separately for participants with lower and higher empathy levels by keeping the respective SPF subscale score constant at high levels (i.e., *M* + 1 *SD*) and at low levels (i.e., *M* – 1 *SD*). In the case of multiple comparisons, uncorrected *p*-values are reported. The risk of α error accumulation is addressed in the discussion.

**Table 3:** Data points per subscale per participant included in the N400 and rating LME analyses.

	Dependent variable											
	N400			Absolute valence			Arousal			Emotional experience		
Subscale	Total	*M* ^a^	*SD* ^a^	Total	*M*	*SD*	Total	*M*	*SD*	Total	*M*	*SD*
Empathic Concern	77268	55.61	3.52	13444	58.96	1.55	13882	59.32	1.69	13814	59.03	2.34
Fantasy	77278	55.61	3.51	13442	58.96	1.55	13875	59.29	1.70	13813	59.03	2.36
Personal Distress	77271	55.61	3.53	13448	58.98	1.54	13876	59.30	1.69	13813	59.03	2.30
Perspective Taking	77265	55.61	3.53	13449	58.99	1.47	13879	59.31	1.67	13814	59.03	2.35

Each analysis included a minimum of ≥ 41 and a maximum of 60 data points per participant per condition (per electrode in the LME analysis on the N400). *M* = mean, *SD* = standard deviation.

^a^*M* and *SD* of data points per participants calculated for electrode FC5.

Additionally, we conducted model comparisons in the form of χ^2^ tests on the loglikelihood ratio of the models including one of the SPF subscales as predictors, respectively, and a base model consisting of only emotionality and laterality as fixed effect factors. Random effect structures were the same as reported above in all models. Model comparisons were used to compare the base model against all four empathy-subscale models per dependent variable in order to estimate explanatory power of each subscale score.

### Rating LME analyses

We set up four separate LME analyses (one per SPF subscale) for each rating scale: absolute valence (0–4, absolute values computed from the bipolar signed valence scale [−4–4]; for an additional analysis of signed valence, see S1 File), arousal (1–9), and emotional experience (1–9). Each model included the fixed-effect predictors emotionality and one of the four empathy measures (as specified for the N400 LME analyses) and the random slopes and intercepts per subject and word, resulting in the following model terms (with “rating” standing for one of the three rating-based dependent variables and “empathy measure” for one of the four subscale scores, respectively):


Rating ~ emotionality * empathy measure + (1|subject) + (1|word)

The rationale behind the definition of the random effect structure was the same as explained above for the N400 analyses. Further, we followed the same procedure of outlier rejection (see Table 3), planned contrasts, and resolution of significant interactions and model comparisons as specified above for the N400 analyses.

## Results

For better readability, we only report uncorrected *p*-values for significant (α = .05) effects in the text, full inferential statistics are displayed in the corresponding tables. A conservative Bonferroni correction would yield an α-level of .017 for contrasts and simple slope analyses (α/3 comparisons) and an α-level of .0125 for model comparisons (α/4 comparisons). The risk of α error accumulation is addressed in the discussion.

### N400

**Fig 2:**
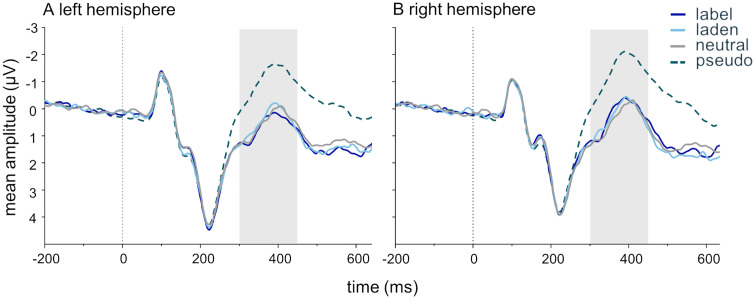
Grand average ERP curves per word type. Grand averages emotionality level pooled over the three left hemispheric (F3, FC1, FC5) and three right hemispheric (F4, FC2, FC6) electrodes for all participants (*n* = 78). Grey areas mark the N400 time window (300-450 ms). Label = emotion-label words, laden = emotion-laden words, neutral = neutral words, pseudo = pseudowords.

Fig 2 shows grand average ERP waveforms for all 78 participants, pooled over the three frontocentral electrodes over the left (FC1, FC5, F3) and the right hemisphere (FC2, FC6, F4), respectively. Note that the interindividual differences introduced by the SPF subscale scores are not displayed. Pseudowords are displayed for validation purposes only and were not included in the analyses.

#### Empathic concern.

**Fig 3:**
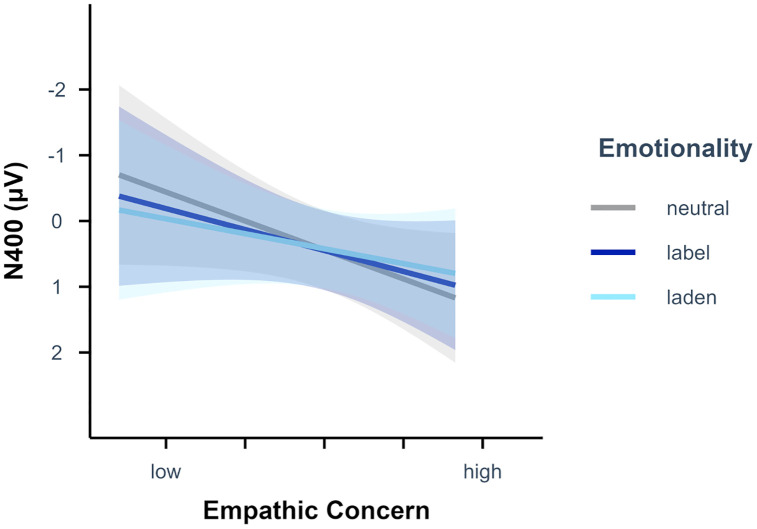
Emotionality-specific effects of empathic concern scores on N400 amplitudes. Empathic concern scores displayed from low (*M* – 2 *SD*) to high (*M +* 2 *SD*) for *n* = 78 participants. Semi-transparent ribbons indicate 90% confidence intervals.

The N400 LME analysis including the empathic concern subscale scores revealed a significant interaction of emotionality and empathic concern, *p* = .006. Simple slope analyses (see Fig 3) with empathic concern as predictor and emotionality as moderator showed that the effect of empathic concern on N400 amplitudes was neither significant for emotion-label, β = 0.32 (*SE* = 0.29), *t* = 1.11, *p* = .270, nor for emotion-laden, β = 0.23 (*SE* = 0.29), *t* = 0.79, *p* = 0.43, nor for neutral words, β = 0.44 (*SE* = 0.29), *t* = 1.53, *p* = .129. Planned contrasts revealed that the effect of empathic concern was significantly stronger for neutral than for emotion-laden words, *p* = .002, while it did not differ significantly between emotion-label and neutral, *p* = .070, and emotion-label and emotion-laden words, *p* = .170. Further exploration of the interaction revealed that the effect of emotionality was neither significant for participants with lower empathic concern scores, *F*(2, 240) = 0.61, *p* = .545, nor for participants with higher empathic concern scores, *F*(2, 240) = 0.85, *p* = .431. No other main or interaction effects in the N400 LME analysis including empathic concern were significant, all *p* ≥ .055 (see Table 4A).

**Table 4:** Inferential statistics for the SPF subscale LME analyses on N400 amplitudes.

Predictors/*Contrasts*	β	*SE*	*df*	*t/F* ^*a*^	*p*
**A. Empathic Concern**					
Emotionality			2, 177	0.01	.985
Laterality	−0.26	0.40	4	−0.65	.550
Empathic Concern	0.33	0.28	76.00	1.16	.251
Emotionality × Laterality			2, 77000	2.90	.055
Emotionality × Empathic Concern			2, 77021	5.07	.006 **
*Emotion-label vs. Neutral*	−0.12	0.07	77020	−1.81	.070
*Emotion-laden vs. Neutral*	−0.21	0.07	77020	−3.18	.002 **
*Emotion-laden vs. Emotion-label*	−0.09	0.07	77020	−1.37	.170
Laterality × Empathic Concern	0.09	0.05	77000	1.65	.099
Emotionality × Laterality × Empathic Concern			2, 77000	0.24	.787
**B. Fantasy**					
Emotionality			2, 177	0.02	.979
Laterality	−0.27	0.40	4	−0.66	.544
Fantasy	0.56	0.28	76.01	2.01	.048 *
Emotionality × Laterality			2, 77010	2.65	.070
Emotionality × Fantasy			2, 77036	15.99	< .001 ***
*Emotion-label vs. Neutral*	0.38	0.07	77040	5.65	< .001 ***
*Emotion-laden vs. Neutral*	0.17	0.07	77030	2.59	.010 **
*Emotion-laden vs. Emotion-label*	−0.21	0.07	77040	−3.06	.002 **
Laterality × Fantasy	0.15	0.05	77010	2.82	.005 **
Emotionality × Laterality × Fantasy			2, 77010	0.10	.908
**C. Personal Distress**					
Emotionality			2, 177	0.02	.984
Laterality	−0.26	0.40	4	−0.66	.545
Personal Distress	−0.05	0.29	76.00	−0.16	.871
Emotionality × Laterality			2, 77003	2.72	.066
Emotionality × Personal Distress			2, 77028	< 0.01	.094
Laterality × Personal Distress	0.00	0.05	77000	−0.05	.959
Emotionality × Laterality × Personal Distress			2, 77003	0.11	.897
**D. Perspective Taking**					
Emotionality			2, 177	0.01	.990
Laterality	−0.26	0.40	4	−0.65	.550
Perspective Taking	−0.10	0.28	76.02	−0.36	.724
Emotionality × Laterality			2, 76997	2.94	.053
Emotionality × Personal Distress			2, 77026	0.34	.710
Laterality × Perspective Taking	−0.04	0.05	77000	−0.76	.446
Emotionality × Laterality × Personal Distress			2, 76997	0.09	.918

Planned contrasts are only shown for significant effects. SPF = Saarbrücker Persönlichkeitsfragebogen; *SE* = standard error; *df* = degrees of freedom.

^a^*t*-statistic for all effects except for main and interaction effects including Emotionality. For effects including Emotionality, *F*-statistic is reported and beta values are not available.

* *p* < .05, ** *p* < .01, *** *p* < .001 (uncorrected)

#### Fantasy.

**Fig 4:**
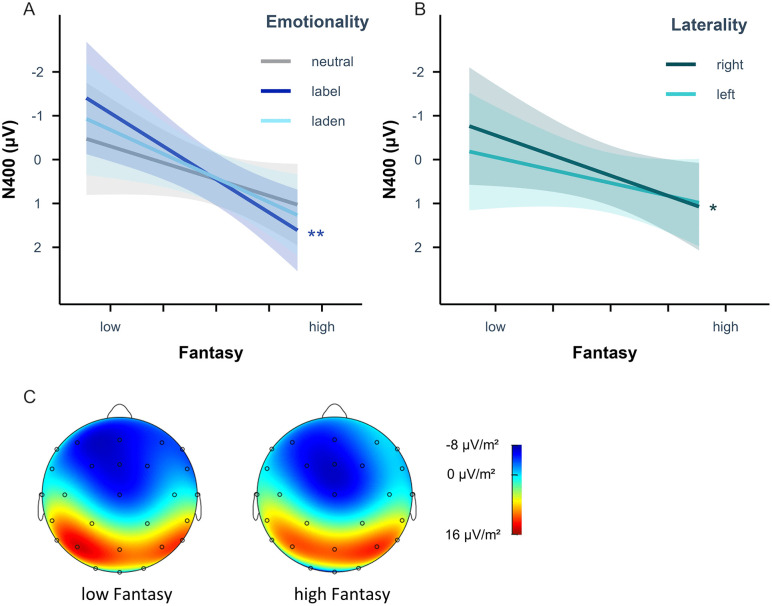
Emotionality-specific (A) and laterality-specific effects (B, C) of fantasy scores on N400 amplitudes. Fantasy scores displayed from low (*M* – 2 *SD*) to high (*M +* 2 *SD*) for emotion-label, emotion-laden and neutral words **(A)** and over the left and right hemisphere **(B)**, for *n* = 78 participants. Semi-transparent ribbons indicate 90% confidence intervals. C. displays topography of current source density over the left and right hemisphere separately for participants with low (*n =* 41 participants) and high fantasy scores (*n =* 37 participants) based on a median split. * *p* < .05, ** *p* < .01 (uncorrected).

The N400 LME analysis including the fantasy subscale scores revealed a significant main effect of fantasy, *p* = .048, in which higher fantasy scores resulted in smaller N400 amplitudes. Further, emotionality and fantasy interacted significantly, *p* < .001. Simple slope analyses with fantasy as predictor and emotionality as moderator (see Fig 4A) showed that the effect of fantasy on the N400 was significant for emotion-label, β = 0.76 (*SE* = 0.28), *t* = 2.69, *p* = .009, but not for emotion-laden, β = 0.55 (*SE* = 0.28), *t* = 1.96, *p* = .054, or for neu*t*ral words, β = 0.38 (*SE* = 0.28), *t* = 1.34, *p* = .185. Planned contrasts revealed that the effect of fantasy was significantly stronger for emotion-label than for neutral words, *p* < .001, for emotion-laden than for neutral words, *p* = .010, and for emotion-label than for emotion-laden words, *p* = .002. Further exploration of the interaction revealed that the effect of emotionality was neither significant for participants with lower fantasy scores, *F*(2, 239) = 2.21, *p* = .112, nor for participants with higher fantasy scores, *F*(2, 239) = 2.29, *p* = .104.

Additionally, laterality and fantasy interacted significantly, *p* = .005. Simple slope analyses with fantasy as predictor and laterality as moderator (see Fig 4B) showed that the effect of fantasy on the N400 was significant over the right hemisphere, β = 0.64, *SE* = 0.28, *t* = 2.28, *p* = .025, but not over the left hemisphere, β = 0.48, *SE* = 0.28, *t* = 1.73, *p* = .088. Fig 4C shows the N400 topography for participants with low and high fantasy scores. No other main and interaction effects in the N400 LME analysis including fantasy were significant, all *p* ≥ .055 (see Table 4B).

#### Personal distress and perspective taking.

The N400 LME analyses including the personal distress (see Table 4C) and the perspective taking subscale score (see Table 4D) did not reveal any significant effects, all *p* ≥ .053.

#### N400 model comparisons.

The model comparisons for the N400 LME analyses revealed that including the empathic concern subscale score, *p* = .035, and the fantasy subscale score, *p* < .001, explained a significant amount of additional variance of N400 amplitudes compared to the base model, whereas the personal distress subscale, *p* = .069, and the perspective taking subscale, *p* = .956, did not (see Table 5). The Akaike information criterion (AIC) preferred the models including the empathic concern and fantasy subscales over the base model and the base model over the personal distress and perspective taking models. The Bayesian information criterion (BIC) always preferred the base model.

**Table 5:** Model comparisons between the models including SPF subscales and the base model.

Model	*n*_par_	AIC	BIC	logLik	*SE*	χ^2 a^	*p*
Base model	10	559500	559593	−279740	559480		
Empathic concern	16	559499	559647	−279733	559467	13.52	.035 *
Fantasy	16	559478	559627	−279723	559446	33.62	< .001 ***
Personal Distress	16	559500	559649	−279734	559468	11.71	.069
Perspective Taking	16	559511	559659	−279739	559479	1.56	.956

SPF = Saarbrücker Persönlichkeitsfragebogen; *n*_par_ = number of parameters; AIC = Akaike information criterion; BIC = Bayesian information criterion; logLik = log-likelihood.

^a^*df* = 6

* *p* < .05, *** *p* < .001 (uncorrected)

### Ratings

#### Absolute valence.

All four LME models on absolute valence ratings including one of the four SPF subscales each revealed a significant main effect of emotionality, all *p* < .001, with absolute valence ratings being significantly larger for emotion-label and emotion-laden than for neutral words as well as for emotion-label than for emotion-laden words, all *p* < .001. In the following, we report additional significant effects for absolute valence ratings from the LME analyses, including the empathic concern, fantasy, and perspective taking subscale scores; including the personal distress subscale score did not reveal any additional significant effects. For complete inferential statistics of the LME analyses on absolute valence see Table 6.

**Table 6:** Inferential statistics for the SPF subscale LME analyses on absolute valence.

Predictors/*Contrasts*	β	*SE*	*df*	*t/F* ^*a*^	*p*
**A. Empathic Concern**					
Emotionality			2, 177	424.13	< .001 ***
*Emotion-label vs. Neutral*	2.12	0.08	177	25.76	< .001 ***
*Emotion-laden vs. Neutral*	2.03	0.08	177.10	24.65	< .001 ***
*Emotion-laden vs. Emotion-label*	−0.09	0.08	177	−1.11	< .001 ***
Empathic Concern	0.12	0.04	73.98	2.89	.005 **
Emotionality × Empathic Concern			2, 13187	53.68	< .001 ***
*Emotion-label vs. Neutral*	0.15	0.02	13190	8.53	< .001 ***
*Emotion-laden vs. Neutral*	0.17	0.02	13190	9.36	< .001 ***
*Emotion-laden vs. Emotion-label*	0.01	0.02	13190	0.83	< .001 ***
**B. Fantasy**					
Emotionality			2, 177	422.15	< .001 ***
*Emotion-label vs. Neutral*	2.12	0.08	177	25.70	< .001 ***
*Emotion-laden vs. Neutral*	2.03	0.08	177.10	24.59	< .001 ***
*Emotion-laden vs. Emotion-label*	−0.09	0.08	177	−1.10	< .001 ***
Fantasy	0.09	0.04	73.99	2.04	.044 *
Emotionality × Fantasy			2, 13185	13.78	< .001 ***
*Emotion-label vs. Neutral*	0.09	0.02	13190	5.04	< .001 ***
*Emotion-laden vs. Neutral*	0.07	0.02	13190	−5.04	< .001 ***
*Emotion-laden vs. Emotion-label*	−0.02	0.02	13190	−1.23	.218
**C. Personal Distress**					
Emotionality			2, 177	420.68	< .001 ***
*Emotion-label vs. Neutral*	2.12	0.08	177	25.65	< .001 ***
*Emotion-laden vs. Neutral*	2.03	0.08	177.10	24.56	< .001 ***
*Emotion-laden vs. Emotion-label*	−0.09	0.08	177	−1.09	< .001 ***
Personal Distress	0.04	0.04	73.98	1.03	.305
Emotionality × Personal Distress			2, 13191	1.40	.248
**D. Perspective Taking**					
Emotionality			2, 177	420.53	< .001 ***
*Emotion-label vs. Neutral*	2.11	0.08	177	25.65	< .001 ***
*Emotion-laden vs. Neutral*	2.02	0.08	177.10	24.55	< .001 ***
*Emotion-laden vs. Emotion-label*	−0.09	0.08	177	−1.10	< .001 ***
Perspective Taking	0.05	0.04	74.04	1.21	.231
Emotionality × Perspective Taking			2, 13193	3.26	.038 *
*Emotion-label vs. Neutral*	0.05	0.02	13190	2.55	.011 *
*Emotion-laden vs. Neutral*	0.02	0.02	13190	1.35	.176
*Emotion-laden vs. Emotion-label*	−0.02	0.02	13190	−1.20	.232

Planned contrasts are only shown for significant effects. SPF = Saarbrücker Persönlichkeitsfragebogen; *SE* = standard error; *df* = degrees of freedom.

^a^*t*-statistic for all effects except for main and interaction effects including Emotionality. For effects including Emotionality, *F*-statistics are reported and beta values are not available.

* *p* < .05, ** *p* < .01, *** *p* < .001 (uncorrected)

**Fig 5:**
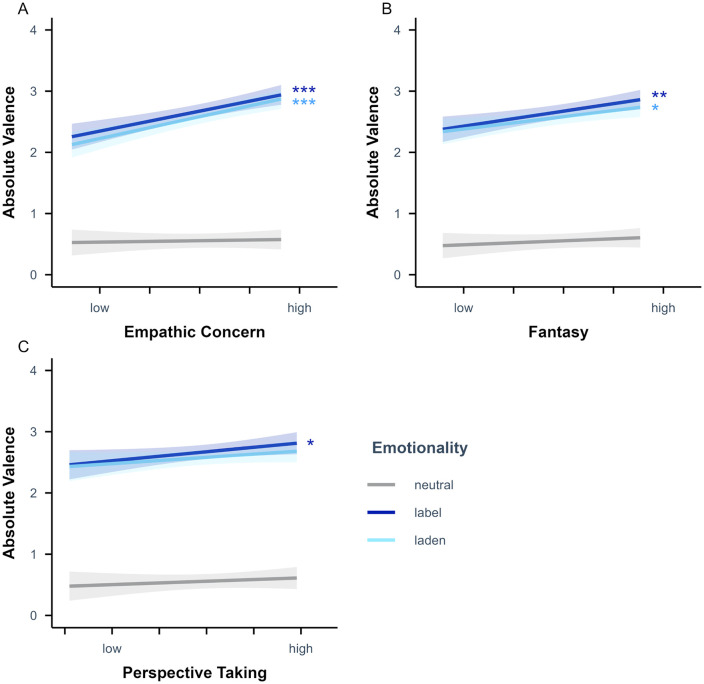
Emotionality-specific effects of (A) empathic concern and (B) fantasy and (C) perspective taking scores on absolute valence ratings. Absolute values were obtained from the Likert scores provided on the bipolar valence scale (i.e., from 0 to | ± 4|). Empathic concern, fantasy and perspective taking values displayed from low (*M* −2 *SD*) to high (*M* + 2 *SD*) for *n* = 76 participants. Semi-transparent ribbons indicate 90% confidence intervals. * *p* < .05, ** *p* < .01, *** *p* < .001 (uncorrected).

The absolute valence rating LME analysis including empathic concern additionally revealed a significant main effect of empathic concern, *p* = .005. Higher empathic concern scores led to higher absolute valence ratings. Further, there was a significant interaction of emotionality and empathic concern, *p* < .001. Simple slope analyses (see Fig 5A) with empathic concern as predictor and emotionality as moderator showed that the effect of empathic concern on the absolute valence ratings was significant for emotion-label, β = 0.16 (*SE* = 0.04), *t* = 3.89, *p* < .001, and for emo*t*ion-laden, β = 0.18 (*SE* = 0.04), *t* = 4.24, *p* < .001, but not for neutral words, β = 0.01 (*SE* = 0.04), *t* = 0.28, *p* = .783. Planned contrasts revealed that the effect of empathic concern on the absolute valence ratings was significantly stronger for emotion-label and emotion-laden than for neutral words as well as for emotion-label than for emotion-laden words, all *p* < .001.

The absolute valence rating LME analysis including fantasy additionally revealed a significant main effect of fantasy, *p* = .044, with valence ratings being higher for higher compared to lower fantasy scores. Further, there was a significant interaction of emotionality and fantasy, *p* < .001. Simple slope analyses (see Fig 5B) with fantasy as predictor and emotionality as moderator showed that the effect of fantasy on the absolute valence ratings was significant for emotion-label, β = 0.12 (*SE* = 0.04), *t* = 2.85, *p* = .006, and for emotion-laden, β = 0.10 (*SE* = 0.04), *t* = 2.34, *p* = .022, but not for neutral words, β = 0.03 (*SE* = 0.04), *t* = 0.76, *p* = .450. Planned contrasts revealed that the effect of fantasy on the absolute valence ratings was significantly stronger for emotion-label and emotion-laden than for neutral words, both *p* < .001, while it did not differ significantly for emotion-label and emotion-laden words, *p* = .218.

The absolute valence rating LME analysis including perspective taking additionally revealed a significant interaction of emotionality and perspective taking, *p* = .038. Simple slope analyses (see Fig 5C) with perspective taking as predictor and emotionality as moderator showed that the effect of perspective taking on the absolute valence ratings was neither significant for emotion-label, β = 0.07 (*SE* = 0.04), *t* = 1.69, *p* = .095, nor for emotion-laden, β = 0.05 (*SE* = 0.04), *t* = 1.20, *p* = .235, nor for neutral words, β = 0.03 (*SE* = 0.04), *t* = 0.64, *p* = .526. Planned contrasts revealed that the effect of perspective taking on the absolute valence ratings was significantly stronger for emotion-label than for neutral words, *p* = .011, while it did not differ significantly for emotion-laden and neutral words, *p* = .176, or for emotion-label and emotion-laden words, *p* = .232.

#### Arousal.

All four LME models on arousal ratings including one of the four SPF subscales each revealed significant main effects of emotionality, all *p* < .001, with arousal ratings being significantly larger for emotion-label and emotion-laden than for neutral words as well as for emotion-label than for emotion-laden words, all *p* < .001. In the following, we report additional significant effects on arousal ratings from the LME analyses including the empathic concern and personal distress subscale score; including the other subscale scores did not yield any additional significant effects. For complete inferential statistics of the LME analyses on absolute valence see Table 7.

**Table 7:** Inferential statistics for the SPF subscale LME analyses on arousal.

Predictors/*Contrasts*	β	*SE*	*df*	*t/F* ^*a*^	*p*
**A. Empathic Concern**					
Emotionality			2, 177	252.45	< .001 ***
*Emotion-label vs. Neutral*	3.18	0.15	177.00	20.98	< .001 ***
*Emotion-laden vs. Neutral*	2.65	0.15	176.90	17.46	< .001 ***
*Emotion-laden vs. Emotion-label*	−0.53	0.15	177	−3.53	< .001 ***
Empathic Concern	0.36	0.12	76.01	2.99	.004 **
Emotionality × Empathic Concern			2, 13623	11.24	< .001 ***
*Emotion-label vs. Neutral*	0.08	0.04	13620	2.06	.040 *
*Emotion-laden vs. Neutral*	0.19	0.04	13620	4.73	< .001 ***
*Emotion-laden vs. Emotion-label*	0.11	0.04	13620	2.67	.008 **
**B. Fantasy**					
Emotionality			2, 176.90	250.88	< .001 ***
*Emotion-label vs. Neutral*	3.18	0.15	176.90	20.91	< .001 ***
*Emotion-laden vs. Neutral*	2.65	0.15	176.90	17.41	< .001 ***
*Emotion-laden vs. Emotion-label*	−0.53	0.15	177	−3.50	< .001 ***
Fantasy	0.09	0.13	76.01	0.68	.499
Emotionality × Fantasy			2, 13616.20	0.38	.685
**C. Personal Distress**					
Emotionality			2, 176.90	251.08	< .001 ***
*Emotion-label vs. Neutral*	3.18	0.15	176.90	20.92	< .001 ***
*Emotion-laden vs. Neutral*	2.65	0.15	176.90	17.41	< .001 ***
*Emotion-laden vs. Emotion-label*	−0.53	0.15	177	−3.51	< .001 ***
Personal Distress	−0.06	0.13	76.01	−0.43	.668
Emotionality × Personal Distress			2, 13617.20	3.91	.020 *
*Emotion-label vs. Neutral*	−0.06	0.04	13620	−1.53	.125
*Emotion-laden vs. Neutral*	0.05	0.04	13620	1.27	.205
*Emotion-laden vs. Emotion-label*	0.11	0.04	13620	2.79	.005 **
**D. Perspective Taking**					
Emotionality			2, 176.90	250.70	< .001 ***
*Emotion-label vs. Neutral*	3.18	0.15	176.90	20.90	< .001 ***
*Emotion-laden vs. Neutral*	2.65	0.15	176.90	17.42	< .001 ***
*Emotion-laden vs. Emotion-label*	−0.53	0.15	177	−3.48	< .001 ***
Perspective Taking	0.13	0.13	76.02	1.03	.309
Emotionality × Personal Distress			2, 13620.50	0.13	.880

Planned contrasts are only shown for significant effects. SPF = Saarbrücker Persönlichkeitsfragebogen; *SE* = standard error; *df* = degrees of freedom.

^a^*t*-statistic for all effects except for main and interaction effects including Emotionality. For effects including Emotionality, *F*-statistics are reported and beta values are not available.

* *p* < .05, ** *p* < .01, *** *p* < .001 (uncorrected)

**Fig 6:**
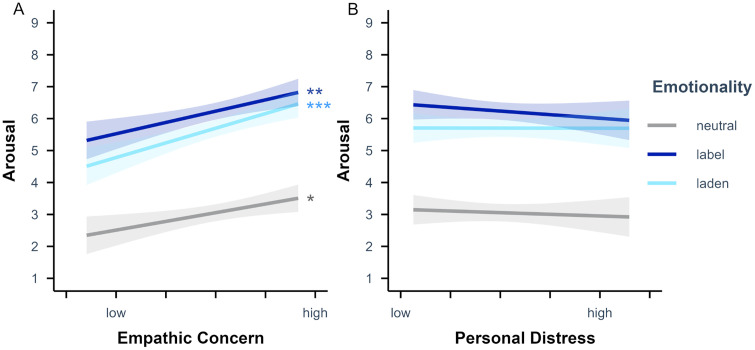
Emotionality-specific effects of (A) empathic concern and (B) personal distress scores on arousal ratings. Values were obtained as Likert scores on the arousal scale (i.e., from 1 to 9). Empathic concern and personal distress values displayed from low (*M* – 2 *SD*) to high (*M +* 2 *SD*) for *n* = 78 participants. Semi-transparent ribbons indicate 90% confidence intervals. * *p* < .05, ** *p* < .01, *** *p* < .001 (uncorrected).

The arousal rating LME analysis including empathic concern additionally revealed a significant main effect of empathic concern, *p* = .004, with higher arousal ratings for higher versus lower empathic concern scores. Further, there was a significant interaction of emotionality and empathic concern, *p* < .001. Simple slope analyses (see Fig 6A) with empathic concern as predictor and emotionality as moderator showed that the effect of empathic concern on the arousal ratings was significant for emotion-label, β = 0.35 (*SE* = 0.12), *t* = 2.87, *p* = .005, emotion-laden, β = 0.46 (*SE* = 0.12), *t* = 3.73, *p* < .001, and neutral words, β = 0.27 (*SE* = 0.12), *t* = 2.21, *p* = .030. Planned contrasts revealed that the effect of empathic concern on the arousal ratings was significantly stronger for emotion-label than for neutral words, *p* = .040, for emotion-laden than for neutral words, *p* < .001, and for emotion-laden than for emotion-label words, *p* = .008.

The arousal rating LME analysis including the personal distress subscale scores additionally revealed a significant interaction of emotionality and personal distress, *p* = .020. Simple slope analyses (see Fig 6B) with personal distress as predictor and emotionality as moderator showed that the effect of personal distress on the arousal ratings was neither significant for emotion-label, β = −0.11 (*SE* = 0.13), *t* = −0.86, *p* = .392, nor for emotion-laden, β < −0.01 (*SE* = 0.13), *t* = −0.02, *p* = .988, nor for neutral words, β = −0.05 (*SE* = 0.13), *t* = −0.40, *p* = .692. Planned contrasts revealed that the effect of personal distress on the arousal ratings was significantly stronger for emotion-label than for emotion-laden words, *p* = .005, while it did not differ between emotion-label and neutral words, *p* = .125, and between emotion-laden and neutral words, *p* = .205.

#### Emotional experience.

All four LME models on emotional experience ratings including one of the four SPF subscales each revealed significant main effects of emotionality, all *p* < .001. Planned contrasts showed that the emotional experience ratings were significantly larger for emotion-label and emotion-laden than for neutral words, as well as for emotion-label than for emotion-laden words, all *p* < .001. In the following, we report additional significant effects on emotional experience ratings from the LME analyses including the empathic concern and personal distress subscale score; including the other subscale scores did not yield any additional significant effects. For complete inferential statistics of the LME analyses on absolute valence see Table 8.

**Table 8:** Inferential statistics for the SPF subscale LME analyses on emotional experience.

Predictors/*Contrasts*	β	*SE*	*df*	*t/F* ^*a*^	*p*
**A. Empathic Concern**					
Emotionality			2, 177	311.68	< .001 ***
*Emotion-label vs. Neutral*	3.88	0.17	177	23.54	< .001 ***
*Emotion-laden vs. Neutral*	3.13	0.17	177	18.98	< .001 ***
*Emotion-laden vs. Emotion-label*	−0.75	0.17	177.10	−4.55	< .001 ***
Empathic Concern	0.25	0.11	76.02	2.21	.030 *
Emotionality × Empathic Concern			2, 13556	12.96	< .001 ***
*Emotion-label vs. Neutral*	0.06	0.04	13560	1.69	.091
*Emotion-laden vs. Neutral*	0.18	0.04	13560	5.01	< .001 ***
*Emotion-laden vs. Emotion-label*	0.12	0.04	13560	3.30	< .001 ***
**B. Fantasy**					
Emotionality			2, 177	312.68	< .001 ***
*Emotion-label vs. Neutral*	3.88	0.16	177	23.56	< .001 ***
*Emotion-laden vs. Neutral*	3.13	0.16	177	19.01	< .001 ***
*Emotion-laden vs. Emotion-label*	−0.75	0.16	177.10	−4.55	< .001 ***
Fantasy	0.11	0.12	76	0.94	.349
Emotionality × Fantasy			2, 13554	1.64	.195
**C. Personal Distress**					
Emotionality			2, 177	312.96	< .001 ***
*Emotion-label vs. Neutral*	3.89	0.16	177	23.59	< .001 ***
*Emotion-laden vs. Neutral*	3.13	0.16	177	19.01	< .001 ***
*Emotion-laden vs. Emotion-label*	−0.75	0.16	177.10	−4.58	< .001 ***
Personal Distress	0.00	0.12	76	0.00	.999
Emotionality × Personal Distress			2, 13554	6.43	.002 **
*Emotion-label vs. Neutral*	0.08	0.04	13550	2.10	.036 *
*Emotion-laden vs. Neutral*	0.13	0.04	13550	3.57	< .001 ***
*Emotion-laden vs. Emotion-label*	0.05	0.04	13550	1.47	.142
**D. Perspective Taking**					
Emotionality			2, 177	312.61	< .001 ***
*Emotion-label vs. Neutral*	3.89	0.16	177	23.57	< .001 ***
*Emotion-laden vs. Neutral*	3.13	0.16	177	19.01	< .001 ***
*Emotion-laden vs. Emotion-label*	−0.75	0.16	177.10	−4.56	< .001 ***
Perspective Taking	0.22	0.11	76.01	1.91	.060
Emotionality × Personal Distress			2, 13556	1.07	.343

Planned contrasts are only shown for significant effects. SPF = Saarbrücker Persönlichkeitsfragebogen; *SE* = standard error; *df* = degrees of freedom.

^a^*t*-statistic for all effects except for main and interaction effects including Emotionality. For effects including Emotionality, *F*-statistics were reported and beta values are not available.

* *p* < .05, ** *p* < .01, *** *p* < .001 (uncorrected)

**Fig 7:**
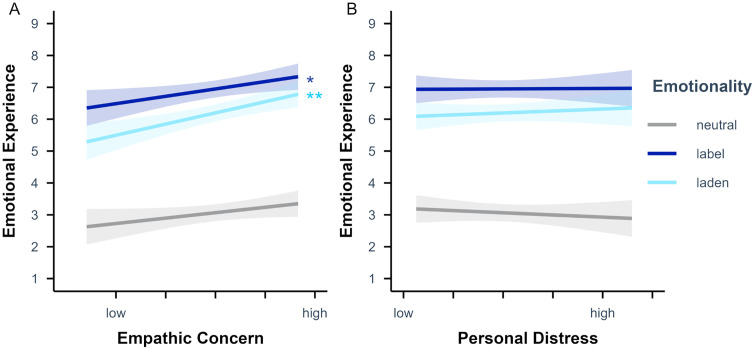
Emotionality-specific effects of (A) empathic concern and (B) personal distress scores on emotional experience ratings. Values were obtained as Likert scores on the emotional experience scale (i.e., from 1 to 9). Empathic concern and personal distress values displayed from low (*M* – 2 *SD*) to high (*M +* 2 *SD*) for *n* = 78 participants. Semi-transparent ribbons indicate 90% confidence intervals. * *p* < .05, ** *p* < .01, *** *p* < .001 (uncorrected).

The LME analysis on emotional experience ratings including empathic concern additionally revealed a significant main effect of empathic concern, *p* = .030. Higher empathic concern scores led to higher emotional experience ratings. Further, there was a significant interaction of emotionality and empathic concern, *p* < .001. Simple slope analyses (see Fig 7A) with empathic concern as predictor and emotionality as moderator showed that the effect of empathic concern on the emotional experience ratings was significant for emotion-label, β = 0.23 (*SE* = 0.12), *t* = 2.01, *p* = .048, and for emotion-laden, β = 0.35 (*SE* = 0.12), *t* = 3.04, *p* = .003, but not for neutral words, β = 0.17 (*SE* = 0.12), *t* = 1.47, *p* = .144. Planned contrasts revealed that the effect of empathic concern on the emotional experience ratings was significantly stronger for emotion-laden than for neutral words, *p* < .001, and for emotion-label than for emotion-laden words, *p* < .001, while it did not differ between emotion-label and neutral words, *p* = .091 (see Table 8A).

The emotional experience rating LME analysis including the personal distress subscale scores additionally revealed a significant interaction of emotionality and personal distress, *p* = .002. Simple slope analyses (see Fig 7B) with personal distress as predictor and emotionality as moderator showed that the effect of personal distress on the emotional experience ratings was neither significant for emotion-label, β = 0.01 (*SE* = 0.12), *t* = 0.06, *p* = .951, nor for emotion-laden, β = 0.06 (*SE* = 0.12), *t* = 0.51, *p* = .613, nor for neutral words, β = −0.07 (*SE* = 0.12), *t* = −0.58, *p* = .567. However, descriptively, higher personal distress scores led to higher emotional experience ratings for emotion-laden words and to lower emotional experience ratings for neutral words (note the change in signs of the respective β estimate). Planned contrasts revealed that the effect of personal distress on the emotional experience ratings differed significantly between emotion-label and neutral words, *p* = .036, as well as between emotion-laden and neutral words, *p* < .001, while it did not differ significantly between emotion-label and emotion-laden words, *p* = .142.

#### Ratings model comparisons.

The model comparisons for the absolute valence rating LME analyses revealed that including the empathic concern subscale score, the fantasy subscale score, and the perspective taking score explained a significant amount of additional variance of absolute valence ratings compared to the base model, all *p* < .001. The personal distress subscale did not explain a significant amount of additional variance, *p* = .374 (see Table 9A).

**Table 9:** Model comparisons between the models including SPF subscales and the base model.

Model	*n*_par_	AIC	BIC	logLik	*SE*	χ^2 a^	*p*
**A.** **Absolute Valence**							
Base model	6	36988	37033	−18488	36976		
Empathic concern	9	36928	36996	−18455	36910	65.71	< .001 ***
Fantasy	9	36972	37039	−18477	36954	22.08	< .001 ***
Personal Distress	9	36991	37058	−18486	36973	3.12	.374
Perspective Taking	9	36977	37044	−18479	36959	17.08	.001 **
**B.** **Arousal**							
Base model	6	59862	59907	−29925	59850		
Empathic concern	9	59842	59910	−29912	59824	25.69	< .001 ***
Fantasy	9	59865	59933	−29924	59847	2.89	.409
Personal Distress	9	59864	59932	−29923	59846	4.16	.245
Perspective Taking	9	59866	59934	−29924	59848	1.95	.582
**C. Emotional Experience**							
Base model	6	58164	58209	−29076	58152		
Empathic concern	9	58154	58222	−29068	58136	15.24	.002 **
Fantasy	9	58166	58234	−29074	58148	4.10	.250
Personal Distress	9	58158	58226	−29070	58140	11.93	.008 **
Perspective Taking	9	58166	58234	−29074	58148	4.11	.250

SPF = Saarbrücker Persönlichkeitsfragebogen; *n*_par_ = number of parameters; AIC = Akaike information criterion; BIC = Bayesian information criterion; logLik = log-likelihood. In all model comparison, the AIC preferred the models explaining a significant amount of additional variance over the base models. The BIC always preferred the base models.

^a^*df* = 3. ** *p* < .01, *** *p* < .001 (uncorrected)

The model comparisons for the arousal rating LME analyses revealed that including the empathic concern subscale score explained a significant amount of additional variance of arousal ratings compared to the base model, *p* < .001. Including the other subscales did not explain a significant amount of additional variance, all *p* ≥ .245 (see Table 9B).

The model comparisons for the emotional experience rating LME analyses revealed that including the empathic concern subscale score, *p* = .002, and the personal distress subscale score, *p* = .008, explained a significant amount of additional variance of absolute valence ratings compared to the base model. Including the fantasy and perspective taking subscale did not explain a significant amount of additional variance, both *p* = .250 (see Table 9C).

## Discussion

This study aimed to uncover differences in the semantic retrieval processes and emotionality-derived representational content between emotion-label, emotion-laden, and neutral abstract words and a differential modulation thereof by specific aspects of empathy, i.e., empathic concern, fantasy, personal distress, and perspective taking. Unexpectedly, we did not find evidence for stand-alone N400 emotionality effects in the direct comparison of emotion-label, emotion-laden, and neutral words. In line with our hypotheses, the empathic concern and fantasy subscales added explanatory power to the N400 analysis and significantly interacted with word emotionality. Unexpectedly, however, higher empathic concern scores reduced N400 amplitudes more strongly for neutral than for emotion-laden words. In line with our hypotheses, we found a gradual fantasy-driven N400 reduction, which was stronger for emotion-label than for emotion-laden than for neutral words. Notably, this effect of fantasy was significant only for emotion-label words and – irrespective of the word’s emotionality – over the right hemisphere. Regarding the words’ representational content, we found that higher empathic concern and fantasy scores led to higher absolute valence ratings specifically for emotion-label and emotion-laden words, while empathic concern additionally led to higher emotional experience ratings specifically for emotion-label and emotion-laden words as well as higher arousal ratings irrespective of the words’ emotionality. These significant effects were mirrored in an added explanatory power of the two empathy measures for the analyses of the respective ratings. Personal distress and perspective taking did not have any significant modulatory effect on the N400 amplitude and rather weak and inconsistent effects on the ratings.

Our null-findings regarding N400 differences between emotion-label, emotion-laden, and neutral words contrasts with previously reported emotionality effects with reduced N400 amplitudes reflecting facilitated processing for emotional versus neutral words [18,19,30,31] as well as with more fine-grained processing differences between emotion-label and emotion-laden words [30,31]. In line with our results, several other studies did not find evidence for an N400 difference between emotional and neutral words [30,35] or emotion-label and emotion-laden words [19]. Resolutions of the significant interactions between emotionality and the subscale scores for empathic concern as well as fantasy further ruled out empathy-moderated emotionality effects, as they provided no evidence for empathy level-specific emotionality effects (and neither did explorative resolutions of non-significant interactions with personal distress and perspective taking; all *p* ≥ .696). Substantiating the emotionality null effect beyond non-significance, we obtained BIC values from model comparisons of the full models described above and models excluding the emotionality main effect as predictor. The difference in BIC values [65] suggested very strong evidence against a stand-alone main effect of emotionality on the N400 in our study.

Notably, our sample size and design should have assured a power of > 75% to uncover a medium effect [66] as has been reported for the N400 emotionality effect by previous studies employing lexical decision tasks (e.g., η² = 0.22 in [19], and ω² = 0.25 in [20]). Further, the delay we introduced before the lexical decisions should have reduced the risk of carry-over effects of emotional to neutral word trials previously discussed to cause behavioral null findings in intermixed designs [27,67]. Additionally, we ruled out potential confounds introduced by the unexpected differences between emotion-label and emotion-laden words revealed by the valence and/or arousal rating analyses based on post-hoc covariate analyses (see below and S2 File). Eventually, any risk of emotionality effects being masked due to multicollinearity with the empathy effects could be ruled out (all variance inflation factors = 1, suggesting essentially zero multicollinearity). In line with this, there were also no significant emotionality effects in explorative post-hoc analyses including only emotionality or emotionality and laterality as well as their interaction as predictors, all *p* > .987. Thus, neither insufficient power, nor carry-over effects, affective confounds or multicollinearity should have contributed to the unexpected null-finding.

An empirically supported post-hoc explanation could lie in the abstractness of our word stimuli. Previous studies reporting N400 emotionality effects for emotional versus neutral German words included concrete and abstract words [18,20,68]. The restriction to abstract words in our study might have led to N400 floor effects, which – taken together with abstract words’ interindividually variable [69] and context-dependent meanings [70] – might have resulted in a suboptimal signal-to-noise ratio for detecting (purely abstract) N400 emotionality effects. Still, given that emotion-label words are abstract per definition, our a priori stimulus selection and matching was necessary to prevent any concreteness-related confounds of the emotionality manipulation [see, e.g., 24,27]. However, speaking against word abstractness contributing to the null finding, another study with purely abstract emotion-label, emotion-laden and neutral words – albeit in Chinese – showed N400 emotionality effects for both types of emotional words compared to neutral words, while reporting no evidence for a more fine-grained emotionality effect between emotion-label and emotion-laden words [19]. Taken together with no evidence for response time differences between processing German emotion-label versus emotion-laden versus neutral abstract words reported in a behavioral lexical decision paradigm [29] and evidence for language-specific emotional word type effects [71], our unexpected null finding might suggest that emotionality effects do not occur in the German language during implicit processing. Whether the presence of emotionality effects depends on a certain concreteness level or the language under investigation, has to be clarified with further research. Acknowledging potential interindividual variability, future studies on abstract emotionality effects should collect concreteness ratings from the experimental sample for validation and statistical control purposes.

Our results seem to provide evidence for a more subtle manifestation of emotional grounding in the observed N400 reductions driven by specific aspects of empathy. This is in line with previous studies reporting, e.g., the Empathizing Questionnaire score to correlate with the magnitude of social congruency-driven N400 effects [37] and an emotional empathy score to correlate with late positive component amplitudes in response to emotional words [35] as well as a behavioral study reporting a comparable effect of the SPF-based empathy score on reaction times in response to emotion-label words [29]. However, regarding the empathic concern subscale, higher scores unexpectedly reduced the N400 more strongly for neutral than for emotion-laden words. Neutral words might have profited from a general positive correlation of reading-related skills and empathy [72]. This positive effect might have been reduced by saliency-driven interference on emotional word processing as has been previously reported in lexical decision tasks [50,73]. The assumed higher salience specifically for participants with higher empathic concern scores is supported by the robustly enhanced affective word ratings and may additionally have been reinforced by the 2:1 ratio of emotional to neutral words in our experiment. However, as this modulation per se was not significant for any emotionality level and confidence intervals largely overlapped, interpreting this finding as an emotionality level-specific effect is only possible to a limited extent. Alternatively, the stronger modulation for neutral words might hint at possible floor effects for emotion-laden words, as the N400 was descriptively lower for emotion-laden than for neutral words in participants with low empathic concern scores, thereby potentially restricting the variance available for further amplitude reductions. It should be noted that the predictive power of empathic concern for variance in N400 amplitudes might be restricted, as the improvement in model fit compared to base model would not withstand Bonferroni correction.

The fantasy subscale in contrast showed a robust improvement in model fit as well as the expected gradual N400 reduction for higher compared to lower scores, which was significantly stronger for emotion-label than for emotion-laden than for neutral words, while the N400 reduction itself was significant only for emotion-label words. Please note that the stronger fantasy-driven N400 reduction for emotion-label than for emotion-laden and for neutral words, as well as the reduction itself for emotion-label words seem robust, as they would outlast conservative Bonferroni correction. In implicit single word processing, a reduced N400 amplitude is thought to reflect a reduced semantic retrieval either because less (multimodal) semantic information is available or because less information has to be retrieved in order to recognize the word as such [12,30,31]. A reduced multimodality seems unlikely, given findings that emotional words are specifically enriched by emotional, interoceptive as well as motoric experiences [16,74]. Thus, the fantasy-driven N400 reduction for emotion-label words might indicate that the higher the participants’ fantasy scores, the more the (direct) emotional content facilitated semantic retrieval. Notably, such an experience-driven facilitation seems to be generalizable across different abstract domains, as there is analogous evidence for a facilitated processing of abstract mathematical words in experienced mathematicians compared to mathematical novices [75].

Our analyses also yielded evidence for higher fantasy scores reducing the N400 amplitudes irrespective of word emotionality over the right but not over the left hemisphere. The right hemisphere is known to be involved in emotional and social information processing as well as empathy [38,76]. Thus, it appears to be sensitive to semantic processes related to emotional experience, which in turn is modulated by an individual’s capacity to imaginatively immerse and mentally simulate emotional experience as represented by the fantasy subscale [34,42]. Notably, the observed fantasy-driven facilitation might have been mediated either by emotional enrichment [18] or alternatively by refined simulation mechanisms, which are central for grounded cognition [2] and – in parts – taken up by the immersion capacity captured by the fantasy subscale. Overall, the reported N400 modulations involving the fantasy subscale score in interaction with word emotionality and hemispheric laterality are in line with an emotional experience-specific grounding of implicit emotion-label word processing.

Regarding the words’ representational content, the analysis of the psycholinguistic ratings revealed higher ratings of absolute valence, arousal, and emotional experience for emotion-label and emotion-laden compared to neutral words; this pattern replicates previous findings on emotionally enriched representations of emotional words [16,50,77]. The expectedly higher emotional experience ratings for emotion-label than for emotion-laden words are further in line with previous findings on measures related to multimodal emotional experience differentiating these word types including interoception ratings [16,77]. In contrast, the obtained higher absolute valence and arousal ratings for emotion-label than for emotion-laden words contradict recent findings that valence and arousal do not differentiate between emotion-laden and emotion-label words [16], however they are in line with a previous behavioral study [29]. The experimental context in our current and previous study with a 2:1 ratio of clearly emotional to clearly neutral words might have led to an attentional bias towards emotionality, making our participants more sensitive to subtle differences in emotional qualities of the words. Further, the rating differences between emotion-label and emotion-laden words might have been introduced by the explicitness of the rating instructions and might therefore be rather epiphenomenal [44]. They thus might not compromise the interpretability of the effects on implicit semantic word processing in the lexical decision task (see, e.g., [26], for independent effects in implicit versus explicit tasks). Still, as stated above, we statistically controlled the potential confound by valence and arousal in post-hoc LME N400 analyses, which replicated and thus validated the inferential pattern reported above (see S2 File).

Regarding differential effects of the SPF subscales, specifically empathic concern and fantasy led to higher subjective ratings of absolute valence for emotion-label and emotion-laden words. This pattern is in line with a behavioral study reporting higher empathy scores (i.e., the sumscore including empathic concern, fantasy, and perspective taking) to lead to significantly higher absolute valence ratings for emotion-label and emotion-laden words, as well as significantly higher arousal and interoception ratings of emotion-label words [29]. Apart from this finding and adding nuance to the previous finding regarding the overall empathy score, empathic concern rather than fantasy seems to have exerted the most extensive and robust effects on the affective ratings, further leading to higher emotional experience ratings specifically for emotion-label and emotion-laden words and higher arousal ratings irrespective of the word emotionality. Perspective taking and personal distress yielded rather inconsistent effects. While the general modulation, i.e., a positive relationship between affective ratings and empathy aspects, has been previously reported specifically for emotional sentences [34], however, this previous result points towards a more extensive influence exerted by perspective taking. This discrepancy might stem from the higher affective and linguistic complexity of sentences compared to the single words used in our study, and sentence processing might have benefitted from the mentalizing abilities going along with perspective taking skills [78].

Taken together, the model comparisons and qualitative comparisons of the inferential pattern of the N400 and rating LME models suggest differential effects of certain empathy aspects captured by the SPF subscales on the semantic processing and representation of specifically emotional words. From the model comparisons, we can conclude that the fantasy scores added robust explanatory power regarding the N400 amplitudes. The absence of evidence for an influence of perspective taking on N400 amplitudes in turn supports the idea that purely cognitive aspects of empathy do not exert a direct impact on the neural mechanisms underlying language processing [37]. Interestingly, we observed that fantasy was the specific aspect of empathy most strongly involved in N400 modulations, while empathic concern showed the strongest effects on the ratings. This might hint at the possibility that the processes involved in implicit and explicit retrieval of conceptual information – as involved in the lexical decision task and ratings, respectively – differ in terms of which specific empathy aspect exerts the strongest influence. It should be noted, that our differential results and the results from a confirmatory factor analysis [41] advocate against an attribution of the SPF subscales to the cognitive versus emotional empathy factor (correlations among the subscales are reported in S3 File).

The specificity indicated by the inferential pattern of the simple slopes in the N400 analyses, especially regarding non-significant findings, must be interpreted with caution due to the relatively narrow range of the obtained SPF subscale scores (see Table 2). Our sample’s demographic characteristics have been shown to favor higher empathy: predominantly female [62 out of 78 participants; 34] young adults [79] studying (mostly) psychology [80,81]. Notably, while the thereby restricted variance might have led to null-findings (see also [34] reporting no correlation of empathy and affective ratings in females), it should not limit the interpretability of the reported significant effects. Still, future studies should include a sample with a wider range of empathy scores while considering additional factors such as sex, gender, age, and profession or field of study in order to confirm emotional experience-driven interindividual differences in N400 modulations that are specific for emotion-label words. Despite this partially limited interpretability of emotionality level-specificity, this study is the first to deliver evidence for empathy-driven interindividual differences in the semantic retrieval and representation of emotion-label, emotion-laden, and neutral abstract word processing to inspire future studies.

## Conclusion

To conclude, our findings suggest that the aspect of empathy involving mental simulation (i.e., fantasy) facilitates the implicit electrophysiological processing of emotion-label words in the lexical decision task. In contrast, the empathy aspect involving prosocial emotional responsiveness (i.e., empathic concern) seems to add to the subjectively perceived emotionality-based richness of emotional word meaning representations visible in the word ratings. These differential effects of empathic concern and fantasy might hint at a dissociation of the underlying mechanisms. Future research can focus on the specific neural and cognitive mechanisms by which certain aspects of empathy influence especially emotion-label words’ grounding in emotional experience. With respect to the theoretical framework, this study provides further evidence that grounding mechanisms can be generalized to the emotional subcategory of abstract concepts, wherein interindividual variability seems to play a crucial role.

## Supporting information

S1 File LME analysis of signed valence ratings. (PDF)

S2 File Signed valence and arousal covariate N400 analyses. (PDF)

S3 File SPF subscale score correlations. (PDF)
