# Supplementary material for: Feel what you read: Specific aspects of empathy modulate semantic retrieval processes and representational content of emotion-label, emotion-laden, and neutral abstract words
Source: PLoS One. 2026 Jan 20;21(1):e0341113. doi: 10.1371/journal.pone.0341113 (PMC12818606; doi:10.1371/journal.pone.0341113)
Supplement: S1 File — (PDF) [file pone.0341113.s001.pdf]

S1: LME analysis of signed valence ratings

In order to test for a potential positivity or negativity bias for our words, we conducted additional LME analyses with signed valence as dependent variable. Otherwise, the analyses were the same as the rating analyses reported in the main text (see Method section). All subscales had a minimum of 49 and a maximum of 60 data points per participants per condition. S1 Table 1 below shows the number of data points included per signed valence LME analysis.

S1 Table 1. Data points per subscale included in the signed valence LME analysis.

Subscale	Total	*M*	*SD*
Empathic Concern	13406	58.80	1.70
Fantasy	13405	58.79	1.70
Personal Distress	13407	58.80	1.69
Perspective Taking	13410	58.82	1.68

M = mean, *SD* = standard deviation.

The signed valence LME analysis did not reveal any significant main or interaction effects, all $p \geq .104$ (see S1 Table 2). Model comparisons (see S1 Table 3) revealed that only the inclusion of personal distress explained a significant additional amount of variance compared to base model, $p = .046$, and was preferred by the AIC. The inclusion of the other SPF subscale scores did not explain additional variance compared to the base model, all $p \geq .282$. The BIC always preferred the base model.

S1 Table 2. Inferential statistics for the SPF subscale LME analyses on signed valence.

Predictors<br>Contrasts	β	*SE*	*df*	*t/F*[a]	*p*
**A. Empathic Concern**					
Emotionality			2, 177	0.27	.764
Empathic Concern	< 0.01	0.03	73.62	-0.15	.879
Emotionality × Empathic Concern			2, 13149.10	1.69	.184
**B. Fantasy**					
Emotionality			2, 177	0.27	.763
Fantasy	0.01	0.03	73.68	0.23	.821
Emotionality × Fantasy			2, 13148.30	0.87	.420
**C. Personal Distress**					
Emotionality			2, 177	0.27	.764
Personal Distress	-0.04	0.02	73.62	-1.65	.104
Emotionality × Personal Distress			2, 13150.10	2.04	.131
**D. Perspective Taking**					
Emotionality			2, 177	0.27	.764
Perspective Taking	-0.01	0.03	73.81	-0.27	.786
Emotionality × Perspective Taking			2, 13153.10	0.51	.601

SPF = Saarbrücker Persönlichkeitsfragebogen; *SE* = standard error; *df* = degrees of freedom. [a]*t*-statistic for all effects except for main and interaction effects including Emotionality. For effects including Emotionality, *F*-statistic is reported and beta values are not available.

S1 Table 3. Model comparisons for the Signed Valence Rating LME analysis.

Model	n_{par}	AIC	BIC	logLik	*SE*	$\chi^{2\,a}$	*p*	
Base model	6	42311	42356	-21149	42299			
Empathic concern	9	42315	42383	-21149	42297	1.33	.722	
Fantasy	9	42313	42381	-21147	42295	3.82	.282	
Personal Distress	9	42309	42376	-21145	42291	7.99	.046	*
Perspective Taking	9	42317	42384	-21149	42299	0.06	.996	

n_{par} = number of parameters; AIC = Akaike information criterion; BIC = Bayesian information criterion; logLik = log-likelihood.
[a] *df* = 3
* *p* < .05 (uncorrected)
