# Supplementary material for: Feel what you read: Specific aspects of empathy modulate semantic retrieval processes and representational content of emotion-label, emotion-laden, and neutral abstract words
Source: PLoS One. 2026 Jan 20;21(1):e0341113. doi: 10.1371/journal.pone.0341113 (PMC12818606; doi:10.1371/journal.pone.0341113)
Supplement: S2 File — (PDF) [file pone.0341113.s002.pdf]

S2: Signed valence and arousal covariate N400 analyses

Method

Inconsistent findings regarding processing differences between emotion-label and emotion-laden words reported in the literature might partially be due to confounding effects of (signed) valence and/or arousal. In order to test whether the N400 LME analysis results reported in the main text are robust against potential confounds of valence and arousal, we conducted an additional LME analysis including the mean centered and normalized signed valence and arousal ratings as well as their interaction with Emotionality as covariates, thereby statistically controlling their potential influence. Apart of that the LME model term was the same as in the main analysis (see Method section; with "empathy measure" standing for one of the four subscale scores, respectively; added fixed-effect covariates highlighted in bold):

*N400 ~ emotionality * laterality * empathy measure* ***+ signed valence + signed valence * emotionality + arousal + arousal * emotionality*** *+ (1|subject) + (1|word) + (1|electrode)*

After residual exclusion, all subscales had a minimum of 42 and a maximum of 60 data points per participants per condition. S2 Table 1 below shows the number of data points included in each covariate analysis.

S2 Table 1. Data points per subscale included in the covariate N400 LME analysis.

Subscale	Total	*M*	*SD*
Empathic Concern	75312	55.62	3.54
Fantasy	75322	55.63	3.52
Personal Distress	75318	55.63	3.54
Perspective Taking	75319	55.64	3.52

M = mean, *SD* = standard deviation.

Results

The covariate analyses N400 LME analyses did not reveal any significant main or interaction effects of signed valence or arousal. Further, the covariate analyses largely confirmed the inferential pattern described in the main analyses: we found significant interactions of emotionality with the empathic concern, $p = .011$, and fantasy subscale, $p < .001$, with the same simple slopes and planned contrasts reaching significance as in the main analyses. The significance of effects in the covariate analyses differed from those in the main analyses only insofar as the main effect of fantasy no longer reached the significance level, $p = .052$. Full inferential statistics of the covariate N400 LME analyses are displayed in S2 Table 2 below.

S2 Table 2. Inferential statistics for the covariate LME analyses on N400 amplitudes

**Predictors** **Contrasts**	**β**	***SE***	***df***	***t/F*[a]**	***p***	
**A. Empathic Concern**						
Emotionality			2, 203	.046	.955	
Laterality	-0.28	0.41	4	-0.69	.527	
Empathic Concern	0.33	0.29	74.05	1.15	.255	
Emotionality × Laterality			2, 75041	2.68	.069	
Emotionality × Empathic Concern			2, 75130	4.55	.011	*
Emotion-label vs. Neutral	-0.11	0.07	75120	-1.65	.099	
Emotion-laden vs. Neutral	-0.21	0.07	75140	-3.01	.003	**
Emotion-laden vs. Emotion-label	-0.09	0.07	75130	-1.37	.170	
Laterality × Empathic Concern	98.05	0.06	75040.00	1.76	.078	
Emotionality × Laterality × Empathic Concern			2, 75041	0.28	.752	
Signed Valence	0.06	0.05	3321	1.14	.254	
Arousal	0.01	0.04	60660	0.20	.842	
Signed Valence × Emotionality			2, 1628	1.17	.311	
Arousal × Emotionality			2, 63676	< 0.01	.995	
**B. Fantasy**						
Emotionality			2, 202	0.10	.904	
Laterality	-0.29	0.41	4	-0.70	.521	
Fantasy	0.57	0.29	74.01	1.98	.052	
Emotionality × Laterality			2, 75051	2.75	.064	
Emotionality × Fantasy			2, 75088	15.27	< .001	***
Emotion-label vs. Neutral	0.38	0.07	75090	5.50	< .001	***
Emotion-laden vs. Neutral	0.15	0.07	75080	2.27	.024	**
Emotion-laden vs. Emotion-label	-0.22	0.07	75090	-3.24	.001	**
Laterality × Fantasy	0.17	0.06	75050	2.92	.003	**
Emotionality × Laterality × Fantasy			2, 75051	0.15	.860	
Signed Valence	0.07	0.05	3333	1.25	.213	
Arousal	0.01	0.04	60800	0.34	.731	
Signed Valence × Emotionality			2, 1641	1.20	.301	
Arousal × Emotionality			2, 64306	0.23	.795	

Predictors<br>Contrasts	β	*SE*	*df*	*t/F*[a]	*p*
**C. Personal Distress**					
Emotionality			2, 203	0.06	.940
Laterality	-0.28	0.41	4	-0.69	.527
Personal Distress	-0.04	0.29	74.00	-0.14	.888
Emotionality × Laterality			2, 75047	-0.69	.072
Emotionality × Personal Distress			2, 75113	-0.14	.179
Laterality × Personal Distress	0.01	0.06	75050.00	0.10	.919
Emotionality × Laterality × Personal Distress			2, 75047	0.07	.930
Signed Valence	0.06	0.05	3319.00	1.19	.235
Arousal	0.01	0.04	60640.00	0.14	.889
Signed Valence × Emotionality			2, 1626	1.21	.298
Arousal × Emotionality			2, 64173	0.17	.844
**D. Perspective Taking**					
Emotionality			2, 203	.071	.931
Laterality	-0.28	0.41	4	-0.69	.526
Perspective Taking	-0.11	0.29	74.02	-0.38	.702
Emotionality × Laterality			2, 75048	2.85	.058
Emotionality × Personal Distress			2, 75087	0.13	.876
Laterality × Perspective Taking	-0.05	0.06	75050.00	-0.85	.395
Emotionality × Laterality × Personal Distress			2, 75048	0.21	.811
Signed Valence	0.06	0.05	3319.00	1.15	.251
Arousal	0.01	0.04	60690.00	0.20	.844
Signed Valence × Emotionality			2, 1630	1.14	.319
Arousal × Emotionality			2, 64120	0.14	.868

Planned contrasts are only shown for significant effects. *SE* = standard error; *df* = degrees of freedom.

[a]*t*-statistic for all effects except for main and interaction effects including Emotionality. For effects including Emotionality, *F*-statistic is reported and beta values are not available.

* $p < .05$, ** $p < .01$, *** $p < .001$ (uncorrected)
