# Supplementary material for: Feel what you read: Specific aspects of empathy modulate semantic retrieval processes and representational content of emotion-label, emotion-laden, and neutral abstract words
Source: PLoS One. 2026 Jan 20;21(1):e0341113. doi: 10.1371/journal.pone.0341113 (PMC12818606; doi:10.1371/journal.pone.0341113)
Supplement: S3 File — (PDF) [file pone.0341113.s003.pdf]

S3: SPF Subscale Score Correlations

We conducted subscale correlation analyses to investigate the interrelationships among the SPF subscales and validate the assumption of higher-order factors of emotional and cognitive empathy. S3 Fig shows the correlations between the scores on the four SPF subscales. Significant correlations were observed between the subscales empathic concern and fantasy, $r = .23$, $p = .040$ (S3 Fig A), empathic concern and personal distress, $r = .25$, $p = .025$ (S3 Fig B), and fantasy and personal distress, $r = .26$, $p = .024$ (S3 Fig C), while none of them correlated significantly with perspective taking (S3 Fig D, E, F), all $r \leq .13$, all $p \geq .247$.

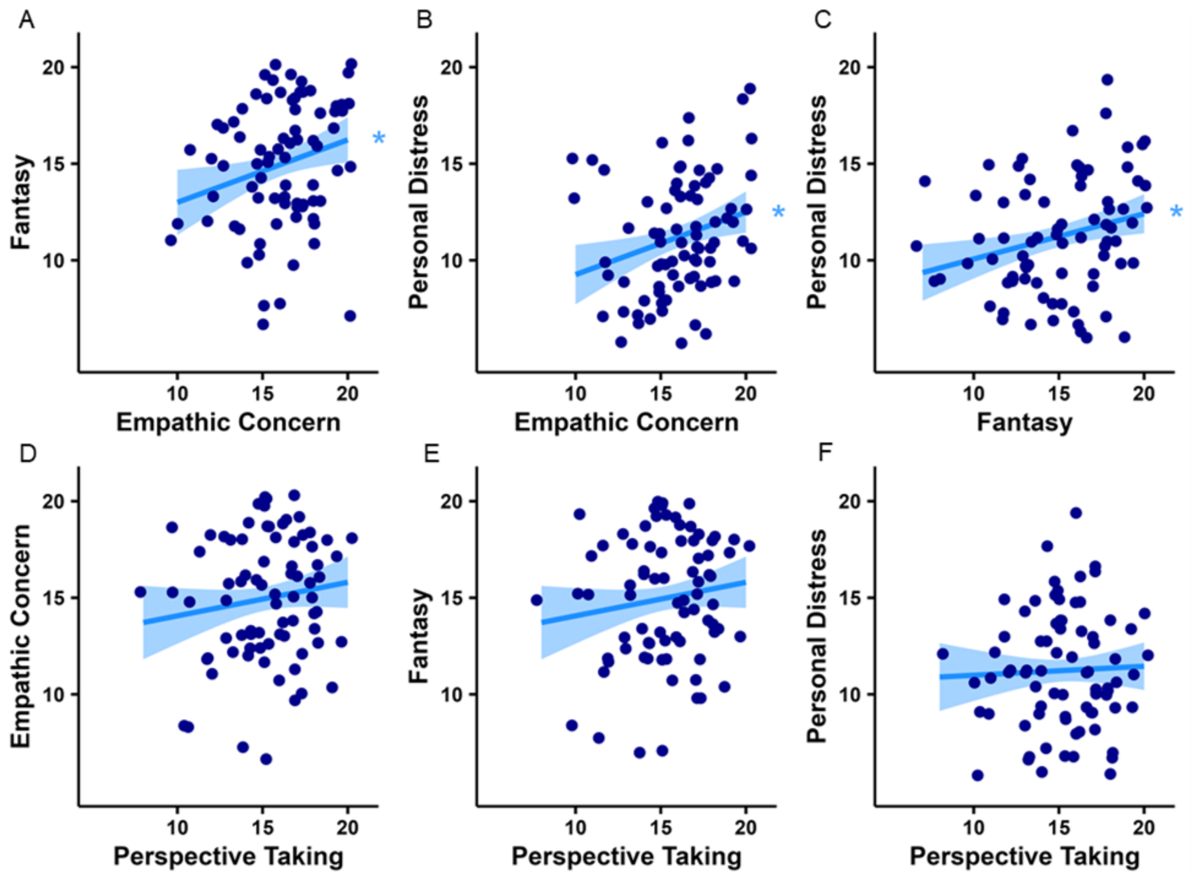

S3 Fig. SPF subscales correlations. SPF = Saarbrücker Persönlichkeitsfragebogen. Axes scaled from lowest to highest reached scores. Data points jittered for better visibility. Semi-transparent ribbons indicate 90% confidence intervals.
* $p < .05$ (uncorrected)
